# Effects of a Multicomponent Lifestyle Intervention on Behavioral and Cardiometabolic Outcomes in Adults with Type 2 Diabetes Mellitus in the Peruvian Amazon

**DOI:** 10.3390/nu18162699

**Published:** 2026-08-18

**Authors:** Mónica Yenji Chia Sánchez, Alexander Javier Iman Torres, Wilson Guerra Sangama, Carlos Antonio Li Loo Kung, Susy Karina Dávila Panduro, Oscar Huaman Gutierrez, Antonio Castillo-Paredes, Jose Jairo Narrea Vargas

**Affiliations:** 1 Departamento de Ciencia y Tecnología de Alimentos, Facultad de Industrias Alimentarias, Universidad Nacional de la Amazonía Peruana, Iquitos 16001, Peru; monica.yenji@gmail.com (M.Y.C.S.); alexander.iman@unapiquitos.edu.pe (A.J.I.T.); 2 Departamento de Ingeniería de Alimentos, Facultad de Industrias Alimentarias, Universidad Nacional de la Amazonía Peruana, Iquitos 16001, Peru; wilson.guerra@unapiquitos.edu.pe (W.G.S.); carlos.li@unapiquitos.edu.pe (C.A.L.L.K.); 3 Departamento de Ciencias Sociales, Facultad de Ciencias de la Educación y Humanidades, Universidad Nacional de la Amazonía Peruana, Iquitos 16000, Peru; susy.davila@unapiquitos.edu.pe; 4 Carrera de Nutrición Humana, Facultad de Medicina, Universidad Nacional Mayor de San Marcos, Lima 15001, Peru; ohuamang@unmsm.edu.pe; 5 Grupo AFySE, Investigación en Actividad Física y Salud Escolar, Escuela de Pedagogía en Educación Física, Facultad de Educación, Universidad de Las Américas, Santiago 8370040, Chile; acastillop85@gmail.com; 6 Grupo de Investigación en Nutrición, Metabolismo y Ejercicio, Carrera de Nutrición y Dietética, Facultad de Ciencias de la Salud, Universidad Científica del Sur, Lima 15067, Peru

**Keywords:** diabetes mellitus, type 2, lifestyle, health behavior, blood glucose

## Abstract

Introduction: Lifestyle interventions are a fundamental component of type 2 diabetes mellitus management; however, evidence from real-world clinical settings regarding multicomponent interventions that simultaneously evaluate behavioral, metabolic, and anthropometric outcomes remains limited. Objective: To evaluate the effect of a multicomponent lifestyle intervention program on dietary habits, biochemical parameters, physical activity, and anthropometric indicators in adults with type 2 diabetes mellitus in the Peruvian Amazon. Methods: A quasi-experimental study with a non-randomized control group and pre–post assessments was conducted among 144 adults with type 2 diabetes mellitus (72 in the intervention group and 72 in the control group) receiving care at a hospital in the Peruvian Amazon. The three-month intervention included nutrition education sessions, practical workshops, group-based physical activity sessions involving traditional dance and Zumba, home visits, and follow-up through telephone calls and WhatsApp. Dietary habits, fasting blood glucose (FBG), glycated hemoglobin (HbA1c), lipid profile, physical activity (PA), body mass index (BMI), waist circumference (WC), and waist-to-height ratio (WHtR) were assessed. Intervention effects were estimated using linear mixed-effects regression models adjusted for age, sex, and disease duration. Results: Compared with the control group, participants receiving the intervention experienced greater improvements in dietary habits (β = 17.85; 95% CI: 16.80 to 18.89; *p* < 0.001), together with greater reductions in FBG (β = −84.23 mg/dL; 95% CI: −100.45 to −68.01; *p* < 0.001) and HbA1c (β = −2.32 percentage points; 95% CI: −2.74 to −1.90; *p* < 0.001). Greater reductions were also observed in triglycerides (β = −53.68 mg/dL), total cholesterol (β = −31.73 mg/dL), BMI (β = −1.67 kg/m^2^), WC (β = −5.31 cm), and WHtR (β = −0.034) (all *p* < file0.001). PA increased to a greater extent among participants receiving the intervention (β = 3015.40 MET-min/week; 95% CI: 2769.81 to 3260.99; *p* < 0.001), whereas no significant between-group difference in change was observed for serum creatinine (*p* = 0.075). Conclusions: Participation in a three-month multicomponent lifestyle intervention was associated with greater improvements in dietary habits, physical activity, glycemic control, lipid profile, and anthropometric indicators compared with usual care among adults with type 2 diabetes mellitus in the Peruvian Amazon.

## 1. Introduction

Type 2 diabetes mellitus (T2DM) is one of the most prevalent chronic diseases worldwide and represents a major public health challenge due to its increasing incidence, long-term complications, and substantial economic burden on healthcare systems. It is characterized by chronic hyperglycemia resulting from insulin resistance and progressive β-cell dysfunction, which contribute to microvascular and macrovascular complications when glycemic control is not adequately achieved [1]. Consequently, maintaining optimal glycemic control remains a primary therapeutic goal.

Although pharmacological treatment plays a crucial role in diabetes care, lifestyle-related factors are important determinants of disease progression and clinical outcomes. Unhealthy dietary habits, physical inactivity, excess adiposity, and poor self-management behaviors have been associated with impaired glycemic control and increased cardiometabolic risk among individuals with T2DM [2,3]. Accordingly, current diabetes management guidelines emphasize the integration of nutritional therapy, regular physical activity (PA), and behavioral interventions into routine clinical care [3].

Lifestyle interventions are a cornerstone of diabetes management and aim to improve patients’ knowledge, skills, and decision-making regarding food choices, dietary patterns, PA, and other self-care behaviors [4]. These interventions may also improve adherence to therapeutic recommendations and facilitate sustainable behavioral changes beyond glycemic management alone [5].

Evidence from systematic reviews and meta-analyses supports the effectiveness of educational and lifestyle interventions in individuals with T2DM. Dietary education interventions have been associated with improvements in glycated hemoglobin (HbA1c), particularly when lasting at least three months and incorporating multiple lifestyle components such as nutrition and PA [6]. Theory- and model-based lifestyle interventions have similarly reduced HbA1c [7], while diabetes education interventions have been associated with improvements in metabolic control, body mass index, and cardiovascular risk indicators, although the magnitude of benefit may vary according to the educational modality [8].

Recent evidence further supports self-management and health education programs in diabetes care. Diabetes self-management education has been associated with improvements in biomedical outcomes, lifestyle behaviors, and anthropometric indicators, particularly when individualized and supported by multidisciplinary healthcare teams [9]. Patient support and educational interventions have also been shown to reduce HbA1c and improve diabetes management outcomes [10], while nutrition counseling and lifestyle intervention programs have reported favorable changes in fasting blood glucose (FBG), HbA1c, dietary practices, and anthropometric parameters [11,12].

Beyond glycemic control, dietary behaviors and PA remain key intervention targets in adults with T2DM. Healthier dietary patterns are associated with improved glucose metabolism and insulin sensitivity, whereas regular PA contributes to weight management, reduced central adiposity, and better metabolic control [13,14]. Nevertheless, adherence to dietary recommendations remains suboptimal in many populations with T2DM, highlighting the need for strategies capable of translating nutritional knowledge into sustainable behavioral changes [15,16].

Despite the well-established benefits of lifestyle interventions for T2DM, evidence regarding their application in real-world clinical settings remains comparatively limited in geographically and culturally distinct populations, including the Peruvian Amazon. The implementation of lifestyle recommendations may be influenced by local food availability, dietary practices, cultural preferences, opportunities for PA, and healthcare resources. Many studies have focused primarily on glycemic markers, whereas fewer have concurrently examined changes in dietary habits, PA, anthropometric indicators, and metabolic outcomes following structured lifestyle interventions [6,9]. Therefore, rather than establishing the general effectiveness of lifestyle modification, context-specific studies are needed to examine how multicomponent programs incorporating locally relevant dietary, physical activity, and behavioral strategies perform when integrated into routine clinical care. Evaluating behavioral, metabolic, and anthropometric outcomes concurrently may provide a broader understanding of their potential application in these settings.

Therefore, the present study aimed to evaluate the effects of a lifestyle intervention program on dietary habits, PA, biochemical parameters, and anthropometric indicators in adults with T2DM in the Peruvian Amazon.

## 2. Materials and Methods

### 2.1. Study Design

This study was conducted as a quasi-experimental, non-randomized controlled trial with a concurrent control group and pre–post assessments. Participants were prospectively assigned to either the lifestyle intervention group or the usual-care control group without randomization. The study was reported in accordance with the Transparent Reporting of Evaluations with Nonrandomized Designs (TREND) statement [17].

### 2.2. Participants

Participants were recruited from the outpatient endocrinology clinic of Hospital III EsSalud Iquitos, located in the province of Maynas, Loreto region, Peru. The study population consisted of 218 patients with a diagnosis of type 2 diabetes mellitus who were registered at the clinic during August 2025.

### 2.3. Sample

A census-based recruitment approach was used, with the aim of including all patients in the target population who met the eligibility criteria. Eligible participants were men and women aged 18–59 years with a diagnosis of type 2 diabetes mellitus, enrolled in the type 2 diabetes care program at Hospital III EsSalud Iquitos, and who provided written informed consent. Patients with three or more comorbidities, those in critical condition, pregnant women, and patients with diabetes-related amputations were excluded. As a result of the participant selection process, the final sample consisted of 145 participants.

Participants were allocated to the study groups using a non-randomized approach based on the appointment schedule previously established by the endocrinology service for August 2025. Random allocation was not implemented because of feasibility constraints within the routine clinical setting. Accordingly, the first 73 eligible patients enrolled according to the pre-existing appointment order were assigned to the intervention group, whereas the subsequent 72 patients were assigned to the control group. The appointment schedule had been established by the clinical service before study enrollment and was not generated or modified by the research team for allocation purposes. No allocation concealment procedure was implemented because group assignment was non-randomized and followed this predefined sequence.

**Figure 1:**
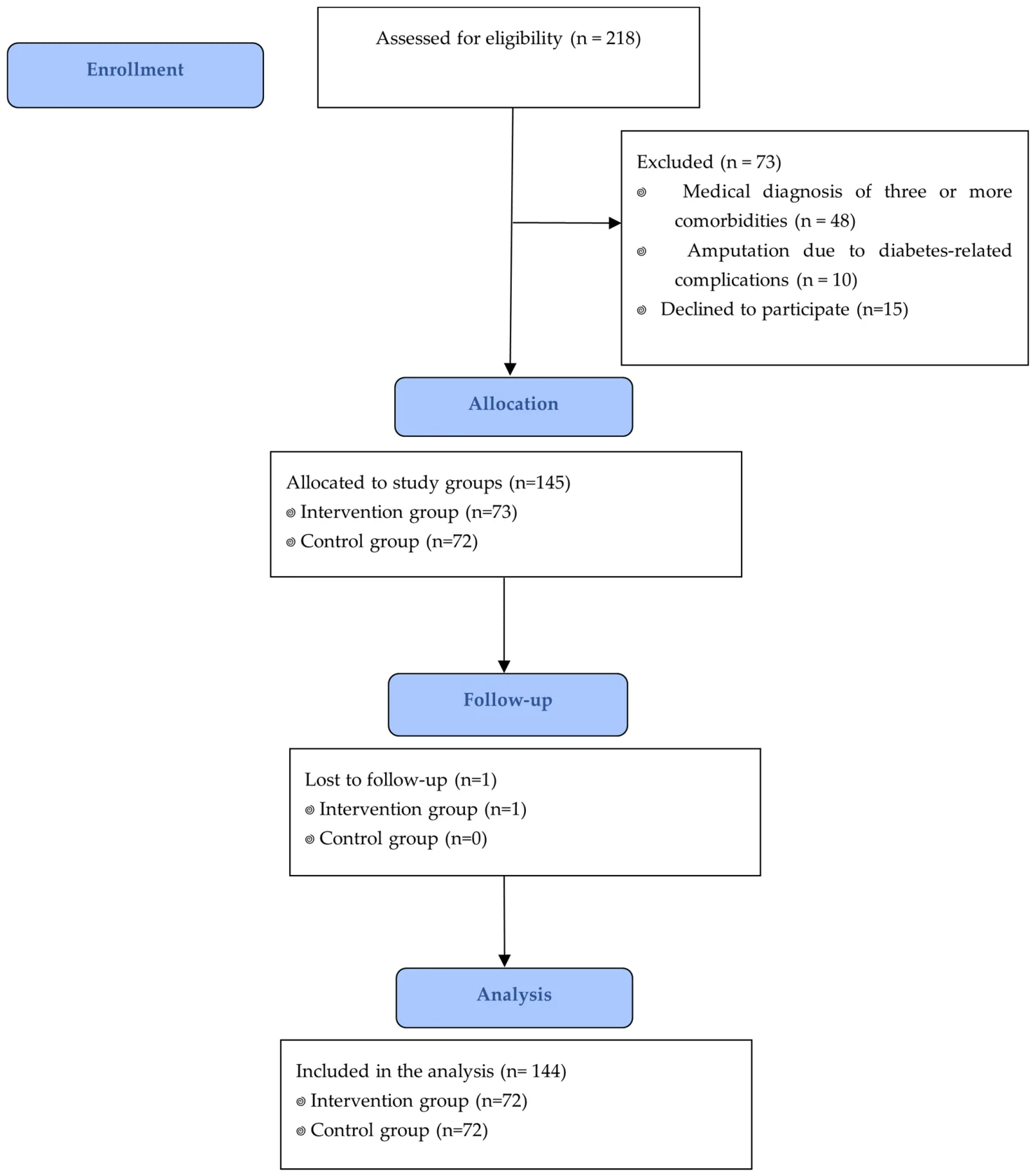
Participant flow diagram according to the TREND statement. The diagram illustrates participant recruitment, allocation to the intervention and control groups, follow-up, and inclusion in the final analysis.

During follow-up, one participant in the intervention group was lost due to death from causes unrelated to the study. Consequently, the final analysis included 144 participants, with 72 patients in the intervention group and 72 patients in the control group (Figure 1).

### 2.4. Intervention Procedures

Before the study commenced, formal contact was established with the administration of Hospital III EsSalud Iquitos through a written request describing the objectives, scope, and procedures of the research. After institutional authorization was obtained, coordination was carried out with the administrative and clinical staff of the endocrinology outpatient clinic to facilitate researchers’ access and the implementation of the planned activities.

Subsequently, face-to-face informational meetings were conducted with eligible patients. During these meetings, the study objectives, assessment procedures, characteristics of the intervention, potential benefits and risks, and the voluntary nature of participation were explained. All participants who agreed to participate provided written informed consent before enrollment.

After informed consent was obtained, baseline assessments were conducted, including anthropometric measurements, biochemical parameter evaluation, dietary habit assessment, and physical activity assessment. Participants assigned to the intervention group then began the lifestyle intervention program.

The intervention lasted three months and included nutrition education sessions aimed at promoting healthy eating habits, as well as supervised group-based physical activity sessions involving traditional dance and Zumba, conducted three times per week. All activities were delivered by trained professionals at the hospital facilities according to a predefined schedule.

The intervention was adapted to the local context by emphasizing affordable and locally accessible foods and meal preparations, incorporating traditional dance as a locally relevant form of PA, and using available healthcare and communication resources, including hospital facilities, home visits, telephone counseling, and WhatsApp-based follow-up.

Follow-up assessments were performed at the end of the intervention period using the same instruments, procedures, and assessment conditions employed at baseline. All assessments and intervention activities were conducted between September and December 2025, from 08:00 to 18:00 h, at Hospital III EsSalud Iquitos.

Throughout the three-month study period, participants in both groups continued to receive their usual diabetes care at the endocrinology outpatient clinic, including their prescribed glucose-lowering treatment with metformin and/or insulin, as applicable. Pharmacological treatment was not discontinued as part of the study, and the lifestyle intervention did not include modifications to glucose-lowering medication. Medication adherence, however, was not systematically assessed.

Participants in the control group did not participate in the lifestyle intervention during the follow-up period and received usual care only. However, for ethical reasons, the same intervention program was offered to and delivered to these participants after completion of the study.

#### 2.4.1. Dietary Habits

Dietary habits were assessed using a Food Frequency Questionnaire, administered before the intervention and again at the end of the three-month follow-up period. The questionnaire was developed based on the structure proposed by Willett [18] and adapted to the local dietary context for the present study.

The adapted version consisted of eight food groups comprising a total of 36 items. Each item was rated using a five-point Likert scale according to consumption frequency: daily, 3–6 times per week, 1–2 times per week, 1–3 times per month, and never. The complete list of foods included in the questionnaire is provided in Supplementary Material File S1.

Foods were classified as healthy or unhealthy according to the recommendations of the Peruvian Dietary Guidelines and the World Health Organization (WHO). Healthy foods included those recommended for regular consumption, such as fruits, vegetables, legumes, whole grains, water, and minimally processed animal-source foods. In contrast, unhealthy foods included sugar-sweetened beverages, ultra-processed foods, salty snacks, confectionery, and foods high in added sugars, saturated fats, or sodium.

For products whose classification was not straightforward, such as flavored yogurts, fortified breakfast cereals, and other mixed processed foods, classification was based on their nutritional profile and degree of processing. Particular consideration was given to the content of added sugars, saturated fats, and sodium, as well as to food classification systems based on nutrient profiling and food processing recommended by international organizations. Products containing substantial amounts of added sugars or classified as ultra-processed were considered unhealthy.

The questionnaire score was calculated using direct scoring for healthy foods and reverse scoring for unhealthy foods, so that a higher total score reflected healthier dietary habits. Total scores ranged from 48 to 168 points and were categorized as unhealthy (48–108 points), moderately healthy (109–138 points), and healthy (139–168 points). These categories and cutoff values were established a priori by the research team as interpretative thresholds for the present study and were not derived from externally validated clinical or diagnostic cutoffs. Accordingly, the continuous total score, rather than these categories, was used as the outcome in the statistical analyses.

Content validity was assessed by five experts with experience in clinical nutrition and public health, yielding an Aiken’s V coefficient of 0.88. A pilot study was also conducted in a population with characteristics similar to those of the study sample, in which the questionnaire showed adequate internal consistency (Cronbach’s α = 0.86). These findings provided evidence of content validity and internal consistency for its use in the present study. However, other measurement properties, including construct validity, criterion validity, test–retest reliability, responsiveness to change, and external validation in an independent population, were not evaluated.

#### 2.4.2. Biochemical Parameters

The biochemical parameters evaluated included FBG, HbA1c, serum creatinine, total cholesterol, and triglycerides. Data were obtained from laboratory reports recorded in each participant’s medical record.

For the purposes of the study and in coordination with the endocrinology outpatient clinic at Hospital III EsSalud Iquitos, participants were scheduled for morning blood sampling under fasting conditions. Biochemical analyses were performed at the hospital’s clinical laboratory at baseline and after three months of follow-up.

All laboratory analyses were carried out by trained laboratory personnel following standardized operating procedures, national quality assurance guidelines for clinical laboratories, and the international quality and competence standards for medical laboratories (ISO 15189) [19].

At each assessment time point, samples from participants in both study groups were analyzed within the same analytical batch; all baseline samples were analyzed together, followed by the analysis of all post-intervention samples.

#### 2.4.3. Physical Activity

Physical activity was assessed using the short version of the International Physical Activity Questionnaire (IPAQ-SF), a seven-item instrument designed to estimate physical activity performed during the previous seven days across different intensity levels (vigorous activity, moderate activity, walking, and sedentary time). The IPAQ-SF has demonstrated adequate reliability and validity for assessing physical activity in adult populations across different countries and is widely used in epidemiological and clinical research [20]. It has also shown satisfactory reproducibility and applicability among individuals with type 2 diabetes mellitus [21].

Results were expressed as MET-minutes per week (MET-min/week), calculated according to the IPAQ scoring protocol by multiplying the duration, frequency, and metabolic equivalent (MET) value assigned to each type of physical activity.

#### 2.4.4. Anthropometric Indicators

Anthropometric assessments were performed by previously trained and standardized researchers under the supervision of an anthropometry specialist, following current Peruvian guidelines [22]. Body weight was measured with participants barefoot and wearing light clothing using a digital scale (Omron HBF-514C, Omron Healthcare Co., Ltd., Kyoto, Japan), with an accuracy of ±1% and automatic calibration. Height was measured using a stadiometer certified by the National Center for Food, Nutrition and Healthy Living (CENAN, Lima, Peru), with an accuracy of 0.1 cm, ensuring correct anatomical positioning of the participant. Waist circumference (WC) was measured using a metallic measuring tape (Lufkin, Apex Tool Group, LLC, Sparks, MD, USA) at the midpoint between the lower margin of the last palpable rib and the top of the iliac crest.

Body mass index (BMI) was calculated as body weight (kg) divided by height squared (m^2^), and the waist-to-height ratio (WHtR) was calculated by dividing WC (cm) by height (cm).

#### 2.4.5. Lifestyle Intervention Program

The Lifestyle Intervention Program was described following the recommendations of the Template for Intervention Description and Replication (TIDieR) checklist to facilitate transparency, reproducibility, and replication of the intervention [23].

The intervention was initiated after completion of the baseline assessments and lasted three months. The program integrated nutrition education and PA promotion and was designed according to the AAMMEE methodology (Analysis, Care, Motivation, Message, Exercise, and Evaluation). Its content was developed in accordance with Peruvian guidelines for the treatment, management, and nutritional care of patients with type 2 diabetes mellitus [24,25].

The educational component consisted of three face-to-face theoretical sessions designed to strengthen participants’ knowledge and self-management skills. Topics included healthy eating for individuals with type 2 diabetes mellitus, carbohydrate counting, and the importance of PA in diabetes management. All educational sessions were delivered by investigator M.C.S. in the hospital auditorium.

The practical component included two face-to-face demonstration workshops focused on applying the knowledge acquired during the educational sessions. These workshops covered the preparation of healthy, low-cost, and locally accessible meals for individuals with type 2 diabetes mellitus and were conducted by investigator M.C.S.

In addition, a supervised PA program consisting of traditional dance and Zumba group sessions was implemented three times per week at the hospital facilities. These sessions were led by a certified instructor with the support of a physiotherapist from the medical center.

Each educational session and practical workshop lasted approximately 60 min. PA sessions lasted between 60 and 90 min, depending on the scheduled activities. Overall, participants received three educational sessions, two practical workshops, and 36 supervised PA sessions, all delivered face-to-face during the intervention period.

To reinforce adherence and promote the maintenance of behavioral changes, a multicomponent follow-up strategy was implemented for participants in the intervention group. This strategy included monthly home visits lasting approximately 20 min per participant, during which educational content was reinforced, participants’ questions were addressed, and individualized recommendations were provided. During these visits, participants also received printed educational materials and a recipe booklet containing healthy recipes adapted to the local context.

Additionally, telephone counseling was provided whenever necessary, and an exclusive WhatsApp group was created for participants in the intervention group. Through this platform, educational materials, motivational videos, and reinforcement messages were shared regularly. The group also facilitated the exchange of experiences, progress, and challenges among participants, fostering peer support and continuous engagement throughout the intervention period.

Attendance at educational sessions, practical workshops, and supervised PA sessions was actively monitored throughout the intervention. Participants who missed a scheduled session were promptly contacted by the study team to identify barriers and encourage continued participation. Participation was additionally facilitated by providing assistance with the scheduling of routine medical appointments and access to prescribed medications within the healthcare facility.

### 2.5. Ethical Considerations

The study was approved by the Institutional Research Ethics Committee of the Loreto Healthcare Network, EsSalud (approval code: No. 000019-CEIN-GRALO-ESSALUD-2025), in accordance with the principles of the Declaration of Helsinki [26] and the Singapore Statement on Research Integrity, which establishes the fundamental principles of responsible research conduct [27]. Written informed consent was obtained from all participants prior to enrollment. Participant confidentiality was ensured throughout the study, and all data were managed in accordance with Peruvian Personal Data Protection Law No. 29733 and its implementing regulations.

### 2.6. Statistical Analysis

Descriptive statistics were used to characterize the study population at baseline. Continuous variables were summarized as means and standard deviations (SD) or medians and interquartile ranges (IQR), according to their distribution, whereas categorical variables were presented as absolute and relative frequencies. The normality of continuous variables was assessed using the Shapiro–Wilk test and graphical inspection of histograms and Q–Q plots.

Baseline comparability between the intervention and control groups was evaluated using the independent-samples Student’s t-test or the Mann–Whitney U test, as appropriate. Categorical variables were compared using Pearson’s chi-square test or the Fisher–Freeman–Halton exact test when expected cell counts were low.

Study outcomes were defined a priori as primary or secondary outcomes. The primary outcomes were dietary habit score, HbA1c, and FBG. Secondary outcomes included PA expressed as MET-minutes per week, triglycerides, total cholesterol, serum creatinine, BMI, WC, and WHtR.

The effect of the lifestyle intervention program on each outcome was evaluated using linear mixed-effects regression models. These models accounted for the longitudinal structure of the data by including a participant-level random intercept to model the correlation between repeated measurements obtained before and after the intervention.

Study group (intervention vs. control), time (post-intervention vs. baseline), and the group × time interaction were included as fixed effects. The group × time interaction was considered the primary parameter of interest because it estimated the differential change attributable to the lifestyle intervention program between the two groups over time.

Linear mixed-effects models were used to estimate between-group differences in changes over time. Participant ID was specified as the subject variable, with a random intercept included to account for within-participant correlation between repeated measurements. A variance components covariance structure was specified for the random effect. Fixed effects included group, time, the group × time interaction, age, sex, disease duration, marital status, and educational level. The group × time interaction was the primary parameter of interest and represented the adjusted between-group difference in pre–post change. Model parameters were estimated using restricted maximum likelihood (REML), with degrees of freedom approximated using the Satterthwaite method. Random slopes for time were explored but were not retained because the corresponding models failed to converge and yielded unstable covariance parameter estimates. Model assumptions were assessed through inspection of normal Q–Q plots of the residuals and residual-versus-fitted plots to evaluate residual normality and homoscedasticity.

Adjusted models additionally included age, sex, disease duration, marital status, and educational level as potential confounding variables. For each outcome, regression coefficients (β), 95% confidence intervals (95% CI), and corresponding *p*-values were reported.

To visualize individual longitudinal changes in the primary outcomes, paired raincloud plots were generated using the ggplot2 package in R (version 4.5.0). Statistical analyses were performed using IBM SPSS Statistics version 25 (IBM Corp., Armonk, NY, USA). A two-sided *p*-value < 0.05 was considered statistically significant.

## 3. Results

Table 1 presents the baseline characteristics of the participants according to study group. No statistically significant between-group differences were observed in the measured clinical, biochemical, anthropometric, dietary habit, or PA variables (*p* > 0.05), and no statistically significant difference was observed in sex distribution (*p* = 0.59). In contrast, differences were observed in marital status (*p* = 0.01) and educational level (*p* = 0.03), with a higher proportion of married participants and individuals with basic education in the intervention group. Given the non-randomized allocation, baseline hypothesis tests were interpreted descriptively and were not considered evidence of baseline equivalence. The observed differences in marital status and educational level were therefore considered as potential confounders and included as covariates in the adjusted analyses.

In the intervention group, participation exceeded 90% across all scheduled intervention components, indicating a high level of engagement throughout the three-month program.

Table 2 summarizes the changes observed in the study outcomes according to study group. Participants in the intervention group showed improvements in dietary habits, accompanied by reductions in the main indicators of glycemic control and cardiometabolic health. The dietary habit score increased (Δ = +18.8), whereas FBG and HbA1c decreased by 78.0 mg/dL and 2.2 percentage points, respectively. In addition, reductions were observed in triglycerides (Δ = −38.0 mg/dL), total cholesterol (Δ = −26.5 mg/dL), BMI (Δ = −1.5 kg/m^2^), WC (Δ = −4.8 cm), and WHtR (Δ = −0.02). PA increased markedly (Δ = +3154.5 MET-min/week), whereas serum creatinine levels remained relatively stable.

In contrast, the control group exhibited only minimal changes across most outcomes, with slight increases in FBG (Δ = +6.1 mg/dL), HbA1c (Δ = +0.1 percentage points), triglycerides (Δ = +13.0 mg/dL), total cholesterol (Δ = +5.3 mg/dL), BMI (Δ = +0.1 kg/m^2^), and WC (Δ = +0.6 cm).

The results of the adjusted linear mixed-effects regression models are presented in Table 3. Estimates from the unadjusted models were similar to those obtained from the adjusted models, indicating that adjustment for age, sex, and disease duration did not substantially alter the effect of the intervention on the study outcomes.

Regarding the primary outcomes, participants receiving the lifestyle intervention experienced a greater increase in dietary habit score compared with the control group (β = 17.85; 95% CI: 16.80 to 18.89; *p* < 0.001), together with greater reductions in FBG (β = −84.23 mg/dL; 95% CI: −100.45 to −68.01; *p* < 0.001) and HbA1c (β = −2.32 percentage points; 95% CI: −2.74 to −1.90; *p* < 0.001).

With respect to the secondary outcomes, significant reductions were observed in triglycerides (β = −53.68 mg/dL; 95% CI: −61.26 to −46.11; *p* < 0.001), total cholesterol (β = −31.73 mg/dL; 95% CI: −35.43 to −28.03; *p* < 0.001), body mass index (BMI) (β = −1.67 kg/m^2^; 95% CI: −1.84 to −1.51; *p* < 0.001), WC (β = −5.31 cm; 95% CI: −5.77 to −4.85; *p* < 0.001), and WHtR (β = −0.034; 95% CI: −0.037 to −0.031; *p* < 0.001). In addition, a significant increase was observed in PA, expressed as MET-minutes per week (β = 3015.40; 95% CI: 2769.81 to 3260.99; *p* < 0.001). In contrast, no statistically significant changes were found in serum creatinine levels (β = −0.008 mg/dL; 95% CI: −0.016 to 0.001; *p* = 0.075).

Among the covariates included in the adjusted models, only disease duration was significantly associated with BMI (β = −0.14; *p* = 0.006), indicating that a longer duration of type 2 diabetes mellitus was associated with lower BMI values. No significant associations were observed between age or sex and this outcome.

**Figure 2:**
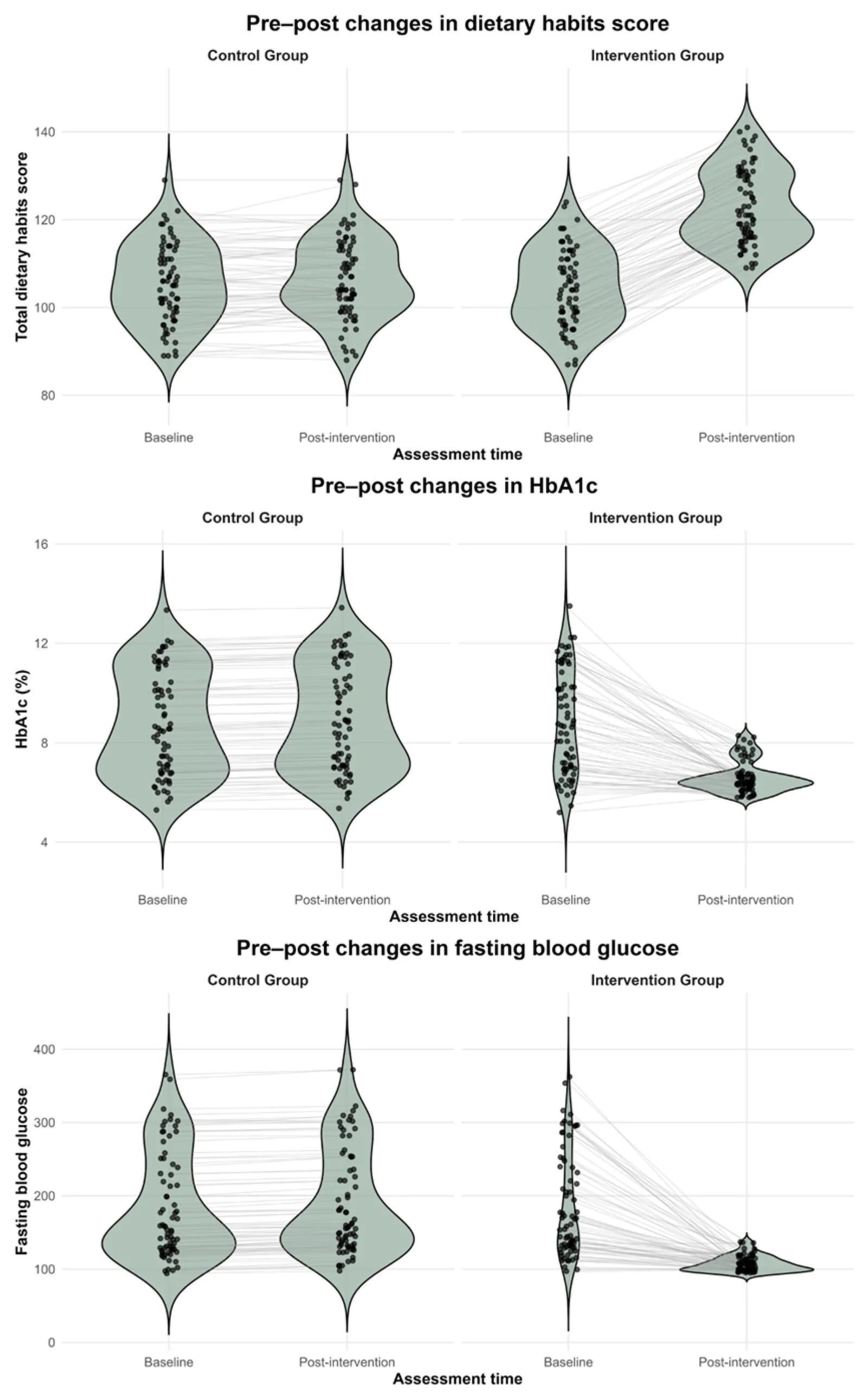
Individual Changes in the Primary Outcomes.

Individual longitudinal changes in dietary habits, FBG, and HbA1c are presented in Figure 2.

## 4. Discussion

The present study evaluated changes in dietary habits, biochemical parameters, PA, and anthropometric indicators following a lifestyle intervention program in adults with type 2 diabetes mellitus. After three months, participants receiving the intervention showed greater improvements in dietary habit scores, FBG, HbA1c, triglycerides, total cholesterol, PA, BMI, WC, and WHtR than those receiving usual care, whereas no significant between-group difference was observed for serum creatinine. Overall, these findings indicate that participation in the multicomponent intervention was associated with simultaneous behavioral, metabolic, and anthropometric improvements, although the magnitude and interpretation of these differences should be considered in light of the non-randomized design.

The reductions observed in HbA1c and FBG are consistent with the general direction of previous studies demonstrating favorable changes in glycemic control following educational and lifestyle interventions in individuals with type 2 diabetes mellitus [28,29,30]. However, the magnitude of the changes observed in the present study was substantially greater than that reported in much of the previous literature. The adjusted between-group differences in change were −2.32 percentage points for HbA1c and −84.23 mg/dL for FBG. In a meta-analysis of randomized controlled trials combining dietary and physical activity interventions, Cradock et al. reported a reduction in HbA1c of −1.11 percentage points at three months and an overall reduction of −0.53 percentage points across follow-up periods [31]. More recently, a network meta-analysis of 108 randomized controlled trials involving 17,735 participants found that diabetes self-management education and support reduced HbA1c by −0.61 percentage points and FBG by −23.33 mg/dL compared with usual care [32]. Thus, although the direction of the glycemic changes observed in our study is consistent with previous evidence, their magnitude was considerably greater. The marked reduction in between-participant variability observed for both HbA1c and FBG at follow-up also indicates a relatively homogeneous glycemic response within the intervention group and warrants cautious interpretation.

Several factors may be relevant when interpreting these comparatively large differences. First, participants in the present study had relatively poor glycemic control at baseline, with a mean HbA1c of approximately 8.8% and FBG of approximately 185 mg/dL, which may have provided greater potential for improvement. This possibility is supported by previous evidence indicating that lifestyle interventions may achieve greater HbA1c reductions among individuals with baseline HbA1c values > 8% [31]. Second, the intervention was intensive and extended beyond nutrition education by incorporating practical workshops, supervised traditional dance and Zumba sessions three times per week, home visits, telephone counseling, and WhatsApp-based support. Previous studies have also suggested that intervention intensity, supervised physical activity, group sessions, and behavioral practice may be associated with greater glycemic improvements [31,33,34,35]. Third, the supervised PA sessions may represent one potential mechanism underlying the observed differences in glycemic and anthropometric outcomes through increased energy expenditure and greater engagement in structured physical activity. Nevertheless, given the non-randomized allocation, the multicomponent nature of the intervention, and the greater contact and attention received by the intervention group, these factors cannot be assumed to explain the large differences observed, nor can the observed changes be attributed to any individual intervention component. The magnitude of the findings should therefore be interpreted cautiously. Although participants in both groups continued their usual glucose-lowering treatment throughout the study, medication adherence was not systematically assessed. Therefore, differences or changes in medication adherence, potentially influenced by the greater contact and follow-up received by the intervention group, cannot be excluded as an additional contributor to the observed glycemic changes and should be considered when interpreting both their magnitude and relative homogeneity.

Participants in the intervention group also showed a marked improvement in dietary habit scores. This behavioral change is relevant because dietary modification was directly targeted through nutrition education, practical workshops, and continued follow-up, providing participants with repeated opportunities to translate nutritional recommendations into everyday food choices. Previous studies have similarly reported improvements in dietary practices following nutrition education and lifestyle interventions in individuals with type 2 diabetes mellitus [36,37,38], including studies conducted in Peru showing improvements in nutritional knowledge, portion size recognition, self-care practices, dietary habits, and adherence to therapeutic recommendations [39,40,41]. In the present study, the improvement in dietary habits may have contributed to the concurrent changes in glycemic and lipid outcomes; however, because dietary intake was self-reported and the intervention combined several behavioral strategies, the extent to which dietary changes independently contributed to these metabolic improvements cannot be determined.

Significant between-group differences were also observed for triglycerides and total cholesterol. These changes occurred alongside improvements in dietary habit scores, PA, and anthropometric indicators, suggesting that the lipid findings should be interpreted within the broader pattern of behavioral and metabolic changes observed during the intervention rather than as an isolated effect. Previous nutrition education and lifestyle interventions have also reported reductions in triglycerides and other lipid parameters [30,37], although findings have not been consistent across studies [29,33,35]. Differences in intervention intensity and the inclusion of structured PA may partly account for this variability. In the present study, however, the multicomponent design prevents determination of whether the observed lipid changes were more closely related to dietary modification, increased PA, changes in adiposity, or their combined influence.

Participants receiving the intervention also showed greater reductions in BMI, WC, and WHtR, indicating that the observed anthropometric changes involved both overall and central adiposity. This pattern is consistent with evidence showing that lifestyle interventions combining nutrition and PA can improve body weight and central adiposity [42], although such effects have not been consistently observed in shorter or less intensive interventions [33,43]. In the present study, the concurrent improvements in dietary habits and PA provide plausible behavioral pathways for the anthropometric changes observed. However, because these components were delivered simultaneously, their individual contributions cannot be separated, and the anthropometric findings should be interpreted as part of the overall response associated with the multicomponent intervention.

The increase in PA was one of the most pronounced findings of the study, with an adjusted between-group difference in change of 3015.40 MET-min/week. Given the magnitude of this difference over only three months, the finding warrants cautious interpretation. The intervention included supervised traditional dance and Zumba sessions three times per week, which may have contributed to greater participation in structured PA; however, PA was assessed using the self-reported IPAQ-SF, which may be susceptible to recall and social desirability biases. In addition, the substantially greater contact received by the intervention group may have enhanced motivation and engagement, and a potential Hawthorne effect cannot be excluded. Thus, the magnitude of the observed increase may reflect a combination of actual changes in PA, self-reporting effects, and greater participant engagement and should not be attributed exclusively to the supervised PA component of the intervention. Previous studies have shown that group-based interventions enhance adherence to PA through social support, motivation, and the establishment of structured exercise routines [44,45]. In addition, greater disease-related knowledge has been associated with better physical activity-related self-care practices among individuals with diabetes [46]. More broadly, participation exceeded 90% across all scheduled intervention components, suggesting that sustained engagement with this intensive multicomponent program was achievable in this clinical setting. However, this high level of participation should be interpreted in the context of active attendance monitoring and prompt follow-up after missed sessions, which may limit the extent to which similar adherence could be expected in settings without these support strategies.

Unlike the other biochemical outcomes, no significant between-group difference was observed for serum creatinine. This finding may reflect the relatively short follow-up period and the limited sensitivity of serum creatinine alone for detecting subtle changes in renal function. Accordingly, the absence of a significant change should not be interpreted as evidence of either improvement or absence of renal effects. Future studies should incorporate more informative renal markers, including estimated glomerular filtration rate (eGFR) and albuminuria, particularly in individuals with type 2 diabetes mellitus.

### 4.1. Practical Implications

From a practical perspective, these findings have important implications for endocrinology services and diabetes care programs. The intervention was implemented within a real-world hospital setting using feasible strategies, including face-to-face educational sessions, demonstration workshops, group-based PA, home visits, and digital support through WhatsApp. This multicomponent approach could be readily adapted by healthcare facilities seeking to strengthen therapeutic education and promote sustainable lifestyle changes. Furthermore, the use of group activities, affordable foods, and culturally appropriate meal preparations may enhance both the acceptability and scalability of the intervention.

### 4.2. Strengths and Limitations

The strengths of this study include the controlled study design, the pre- and post-intervention assessments, the evaluation of multiple behavioral, biochemical, and anthropometric outcomes, and the use of linear mixed-effects models adjusted for potential confounding variables. Furthermore, describing the intervention according to the TIDieR checklist enhances transparency and facilitates replication in other clinical settings. An additional strength is that the study was conducted within a routine healthcare environment, increasing the practical relevance of its findings for the management of patients with type 2 diabetes mellitus.

Nevertheless, several limitations should be acknowledged. First, participants were assigned to the study groups using a non-randomized procedure based on a pre-existing clinical appointment schedule, which may have introduced selection bias and residual confounding despite the comparability of the main clinical characteristics and primary outcomes at baseline; moreover, the possibility that appointment order was associated with unmeasured participant characteristics cannot be excluded. Second, baseline differences in marital status and educational level were observed between groups and may have influenced adherence and responsiveness to the intervention. Third, some variables potentially associated with cardiometabolic outcomes, such as smoking status, alcohol consumption, and other potential confounding factors, could not be included in the adjusted analyses because they were not collected as part of the original study protocol. Therefore, despite adjustment for the available covariates, residual confounding arising from these unmeasured factors cannot be excluded and should be considered when interpreting the observed associations. Fourth, dietary habits and PA were assessed using self-reported instruments, which are susceptible to recall and social desirability biases; additionally, although the dietary habits questionnaire showed adequate content validity and internal consistency, other measurement properties, including construct validity, test–retest reliability, responsiveness to change, and external validation, were not assessed. Therefore, changes in dietary habit scores should be interpreted with caution. Fifth, the three-month follow-up period precludes conclusions regarding the long-term sustainability of the observed changes. Sixth, because the intervention simultaneously incorporated nutrition education, practical workshops, supervised dance and Zumba sessions, home visits, telephone counseling, and WhatsApp-based follow-up, the independent contribution of each component could not be isolated; consequently, it is not possible to determine which component, or combination of components, contributed most to the observed changes, and the findings should be interpreted as reflecting the multicomponent intervention as a whole rather than any individual strategy. Seventh, participants in the intervention group received substantially more contact and attention from the study team than those in the control group through educational sessions, supervised physical activity, home visits, telephone counseling, and WhatsApp-based support; therefore, part of the observed differences may have been related to greater participant engagement, motivation, or awareness of being observed rather than exclusively to the specific content of the intervention. Consequently, a potential Hawthorne effect cannot be excluded, particularly for self-reported behavioral outcomes such as dietary habits and PA, and because the control group received usual care without an attention-matched intervention, the study was unable to distinguish these nonspecific effects from those associated with the intervention components. Finally, patient-centered outcomes such as quality of life and medication adherence, as well as other clinical outcomes including albuminuria, estimated glomerular filtration rate, and intervention-related adverse events, were not systematically evaluated, nor was a formal adjustment for multiple comparisons applied across the secondary outcomes. Therefore, findings related to these outcomes should be interpreted cautiously given the potential for an increased risk of type I error.

### 4.3. Future Perspectives

Future studies should employ randomized designs with longer follow-up periods to evaluate the long-term sustainability of behavioral and clinical improvements and to improve baseline balance between study groups. Particular attention should be given to sociodemographic characteristics, such as educational attainment and marital status, which may influence engagement with and responsiveness to educational and behavioral interventions. Future studies should also systematically collect and account for relevant potential confounding factors associated with cardiometabolic outcomes, including smoking status and alcohol consumption, to minimize residual confounding and improve the robustness of adjusted analyses. It would also be valuable to compare different intervention intensities and delivery modalities, including face-to-face, digital, and hybrid approaches. In addition, future research should incorporate patient-centered outcomes such as quality of life, self-efficacy, treatment adherence, satisfaction with the intervention, and implementation costs. In Latin American and Amazonian settings, future interventions should also consider cultural adaptations, local food availability, barriers to PA, and socioeconomic factors that may influence the effectiveness of lifestyle intervention programs.

## 5. Conclusions

In this non-randomized controlled study, participation in a three-month multicomponent lifestyle intervention was associated with greater improvements in dietary habits, physical activity, glycemic control, lipid profile, and anthropometric indicators compared with usual care among adults with type 2 diabetes mellitus. These findings suggest that multicomponent, context-specific lifestyle interventions may represent a useful strategy for supporting comprehensive diabetes management in real-world clinical settings. However, given the non-randomized design, the observed associations should not be interpreted as definitive evidence of causality.

## Figures and Tables

**Table 1:** Baseline Characteristics of Participants with Type 2 Diabetes Mellitus According to Study Group.

Variable	Unit	Control Group (*n* = 72)	Intervention Group (*n* = 72)	*p*-Value
		Mean ± SD/Median [IQR]		
Age	years	53.7 ± 5.4	53.8 ± 5.6	0.91
Weight	kg	70.6 ± 14.8	70.7 ± 15.4	0.95
Height	Cm	157.2 ± 15.4	157.2 ± 8.8	0.99
Disease duration	years	10.7 ± 7.2	11.1 ± 8.5	0.78
Dietary habits	Score	105.5 ± 9.1	104.3 ± 8.9	0.45
Fasting blood glucose	mg/dL	184.8 ± 72.4	185.2 ± 70.8	0.97
Glycated hemoglobin	%	8.8 ± 2.1	8.8 ± 2.1	0.95
Creatinine	mg/dL	0.62 [0.54–0.88]	0.63 [0.55–0.90]	0.93
Triglycerides	mg/dL	148.5 [119.4–208.0]	146.0 [120.9–206.4]	0.96
Total cholesterol	mg/dL	173.4 ± 35.1	173.8 ± 37.6	0.95
Physical activity	MET-min/week	365.0 [204.8–829.0]	357.0 [132.0–693.0]	0.29
Body mass index	Kg/m^2^	28.4 ± 4.6	28.5 ± 4.7	0.94
Waist circumference	cm	92.1 ± 13.5	92.4 ± 14.6	0.92
Waist-to-height ratio	-	0.59 ± 0.07	0.58 ± 0.08	0.91
		*n* (%)		
Sex				
	Male	25 (34.7)	22 (30.6)	0.59
	Female	47 (65.3)	50 (69.4)	
Marital status				
	Single	26 (36.1)	14 (19.4)	0.01
	Married	32 (44.4)	51 (70.8)	
	Widowed	8 (11.1)	4 (5.6)	
	Divorced	6 (8.3)	3 (4.2)	
Educational level				
	Basic education	34 (47.2)	43 (59.7)	0.03
	Technical higher education	15 (20.8)	19 (26.4)	
	University education	23 (31.9)	10 (13.9)	

Note: Data are presented as mean ± standard deviation (SD) for continuous variables with approximately normal distributions, median [interquartile range (IQR)] for continuous variables with non-normal distributions, and *n* (%) for categorical variables.

**Table 2:** Changes in study outcomes according to study group.

Variable	Group	Baseline	Post-Intervention	Δ
		Mean ± SD/Median [IQR]		
Dietary habits (score)	Control	105.5 ± 9.1	106.3 ± 9.0	+0.8
	Intervention	104.3 ± 8.9	123.1 ± 8.6	+18.8
Fasting blood glucose (mg/dL)	Control	184.8 ± 72.4	190.9 ± 72.4	+6.1
	Intervention	185.2 ± 70.7	107.2 ± 10.7	−78.0
Glycated hemoglobin (%)	Control	8.8 ± 2.1	8.9 ± 2.1	+0.1
	Intervention	8.8 ± 2.1	6.6 ± 0.6	−2.2
Creatinine (mg/dL)	Control	0.62 [0.54–0.88]	0.64 [0.56–0.91]	+0.02
	Intervention	0.63 [0.55–0.90]	0.63 [0.57–0.89]	+0.0
Triglycerides (mg/dL)	Control	148.5 [119.4–208.0]	161.5 [130.3–223.0]	+13.0
	Intervention	146.0 [120.9–206.4]	108.0 [80.6–171.0]	−38.0
Total cholesterol (mg/dL)	Control	173.4 ± 35.1	178.7 ± 35.0	+5.3
	Intervention	173.8 ± 37.6	147.3 ± 31.9	−26.5
Physical activity (MET-min/week)	Control	365.0 [204.8–829.0]	396.0 [146.0–693.0]	+31
	Intervention	357.0 [132.0–693.0]	3511.5 [2463.0–4407.5]	+3154.5
Body mass index (kg/m^2^)	Control	28.4 ± 4.6	28.5 ± 4.6	+0.1
	Intervention	28.5 ± 4.7	27.0 ± 4.9	−1.5
Waist circumference (cm)	Control	92.1 ± 13.5	92.7 ± 13.5	+0.6
	Intervention	92.4 ± 14.6	87.6 ± 14.9	−4.8
Waist-to-height ratio	Control	0.59 ± 0.07	0.58 ± 0.07	−0.01
	Intervention	0.58 ± 0.08	0.56 ± 0.08	−0.02

Note: Data are presented as mean ± standard deviation (SD) for continuous variables with approximately normal distributions and median [interquartile range (IQR)] for continuous variables with non-normal distributions. Δ represents the absolute change (post-intervention−baseline).

**Table 3:** Effect of the Lifestyle Intervention Program on the Evaluated Outcomes: Adjusted Linear Mixed-Effects Regression Models.

Variable	β	SE	95% CI	*p*-Value
Dietary habits (score)	17.85	0.53	16.80 to 18.89	<0.001
Fasting blood glucose (mg/dL)	−84.23	8.21	−100.45 to −68.01	<0.001
Glycated hemoglobin (%)	−2.32	0.21	−2.74 to −1.90	<0.001
Creatinine (mg/dL)	−0.008	0.004	−0.016 to 0.001	0.075
Triglycerides (mg/dL)	−53.68	3.83	−61.26 to −46.11	<0.001
Total cholesterol (mg/dL)	−31.73	1.87	−35.43 to −28.03	<0.001
Physical activity (MET-min/week)	3015.40	124.24	2769.81 to 3260.99	<0.001
Body mass index (kg/m^2^)	−1.67	0.08	−1.84 to −1.51	<0.001
Waist circumference (cm)	−5.31	0.23	−5.77 to −4.85	<0.001
Waist-to-height ratio	−0.034	0.002	−0.037 to −0.031	<0.001

Note: β represents the group × time interaction effect and therefore the adjusted between-group difference in pre–post change. SE, standard error; 95% CI, 95% confidence interval. Linear mixed-effects models included a participant-specific random intercept and were adjusted for age, sex, disease duration, marital status, and educational level.

## Data Availability

The raw data supporting the conclusions of this article will be made available by the authors on request.

## Supplementary Materials

The following supporting information can be downloaded at https://www.mdpi.com/article/10.3390/nu18162699/s1, File S1: Food Frequency Questionnaire—36 Items. File S2: TREND Statement Checklist.

## Abbreviations

The following abbreviations are used in this manuscript:

FBG	Fasting blood glucose
HbA1c	Glycated hemoglobin
PA	Physical activity
BMI	Body mass index
WC	Waist circumference
WHtR	Waist-to-height ratio
T2DM	Type 2 diabetes mellitus
WHO	World Health Organization
IPAQ-SF	The short version of the International Physical Activity Questionnaire
TIDieR	Template for Intervention Description and Replication
AAMMEE	Analysis, Attention, Motivation, Message, Exercise, and Evaluation
