# Supplementary material for: Effects of a Multicomponent Lifestyle Intervention on Behavioral and Cardiometabolic Outcomes in Adults with Type 2 Diabetes Mellitus in the Peruvian Amazon
Source: Nutrients. 2026 Aug 18;18(16):2699. doi: 10.3390/nu18162699 (PMC13516659; doi:10.3390/nu18162699)
Supplement: Supplementary file 1 [file nutrients-18-02699-s001.zip › Supplementary Material File S1. Food Frequency Questionnaire – 36 Items_US.pdf]

Food Consumption Frequency Questionnaire

Participant ID: ……………… Sex: .... Age: ....

Marital status: ……………………… Educational level: …………………

Duration of type 2 diabetes mellitus : ……………………………………………………

Basic housing services: ……………………………………………

Dear Participant: This questionnaire is part of a study designed to gather information about the healthy eating habits you follow on a daily basis. Without further ado, I thank you in advance for your cooperation.

Instructions: Mark with an "x" where you see fit.

FOOD GROUP	No.	FOOD ITEM	CONSUMPTION FREQUENCY				
			Daily	3–6 times/week	1–2 times/week	1–3 times/month	Never
1.Grains	1	Rice	⑤	④	③	②	①
	2	Oats	⑤	④	③	②	①
	3	Barley	⑤	④	③	②	①
	4	Court	⑤	④	③	②	①
	5	Quinoa	⑤	④	③	②	①
	6	Semolina	⑤	④	③	②	①
	7	Wheat	⑤	④	③	②	①
2. Legumes	8	Peas	⑤	④	③	②	①
	9	Corn	⑤	④	③	②	①
	10	Bean	⑤	④	③	②	①
	11	Chickpea	⑤	④	③	②	①
	12	Lentils	⑤	④	③	②	①
3. Root vegetables	13	Sweet potato	⑤	④	③	②	①
	14	Potato	⑤	④	③	②	①
	15	Olluco	⑤	④	③	②	①
	16	Cassava	⑤	④	③	②	①
4. Meat and Eggs	17	Mutton	⑤	④	③	②	①
	18	Pork	⑤	④	③	②	①
	19	Hen	⑤	④	③	②	①
	20	Fish	⑤	④	③	②	①
	21	Turkey	⑤	④	③	②	①
	22	Chicken	⑤	④	③	②	①
	23	Chicken egg	⑤	④	③	②	①
	24	Quail egg	⑤	④	③	②	①
5. Fruits	25	Rich in Vitamin A	⑤	④	③	②	①

	26	Rich in Vitamin C	⑤	④	③	②	①
**6. Vegetables**	27	Rich in Vitamin A	⑤	④	③	②	①
	28	Rich in Vitamin C	⑤	④	③	②	①
**7. Dairy**	29	Fresh milk	⑤	④	③	②	①
	30	Evaporated milk	⑤	④	③	②	①
	31	Yogurt	⑤	④	③	②	①
	32	Cheese	⑤	④	③	②	①
**8. Cured and Canned Meats**	33	Tuna	⑤	④	③	②	①
	34	Hot dog	①	②	③	④	⑤
	35	Ham Party	①	②	③	④	⑤
	36	Other	①	②	③	④	⑤
